# Editorial: Computational Neuroimage Analysis Tools for Brain (Diseases) Biomarkers

**DOI:** 10.3389/fnins.2022.841807

**Published:** 2022-02-18

**Authors:** Diana M. Sima, Meritxell Bach Cuadra, Tim B. Dyrby, Koen Van Leemput

**Affiliations:** ^1^icometrix, Leuven, Belgium; ^2^AI Supported Modelling in Clinical Sciences (AIMS), Vrije Universiteit Brussel, Brussels, Belgium; ^3^Center for Biomedical Imaging (CIBM), Lausanne, Switzerland; ^4^Department of Radiology, Lausanne University Hospital and University of Lausanne, Lausanne, Switzerland; ^5^Signal Processing Laboratory (LTS5), Ecole Polytechnique Fédérale de Lausanne, Lausanne, Switzerland; ^6^Department of Applied Mathematics and Computer Science, Technical University of Denmark, Lyngby, Denmark; ^7^Danish Research Centre for Magnetic Resonance, Centre for Functional and Diagnostic Imaging and Research, Copenhagen University Hospital Amager and Hvidovre, Copenhagen, Denmark; ^8^Department of Health Technology, Technical University of Denmark, Lyngby, Denmark; ^9^Martinos Center for Biomedical Imaging, Massachusetts General Hospital and Harvard Medical School, Boston, MA, United States

**Keywords:** neuroimaging, biomarkers, brain disease, neurodevelopment, deep learning, domain adaptation, brain segmentation, brain connectivity

This Research Topic focuses on recent advances in the field of neuroimaging biomarkers, including novel imaging technologies, image processing, and artificial intelligence approaches, which are pushing forward the achievement of precision medicine. Indeed, brain imaging often visualizes disease effects with greater sensitivity than clinical observation, thus holding great promise to help diagnose patients at the earliest stages of their disease, when treatment is most effective.

To unleash this potential, neuroimaging biomarkers should be proven to be useful, sensitive and reliable. Nowadays, conventional magnetic resonance imaging is complemented by numerous advanced acquisition and processing techniques, aiming to unravel structural and functional brain connectivity and pathological alterations in brain tissue up to the microstructural level.

Current challenges amount not only to designing clinically feasible acquisition protocols and reliable image processing methods, but also integrating the wealth of data that gets collected in different centers and in different neuroimaging domains in a consistent way to permit reuse in new domains. As large-scale and more complex neuroimaging datasets have been or are being collected in heterogeneous ways by various organizations (with the field of Alzheimer's disease as a notable example), Huguet et al. propose core principles to facilitate reusability and data sharing. They implement an ecosystem of modules and tools, including automated quality control, which is suitable for large neuroimaging studies.

However, the benefits of merging different datasets is often counteracted by their heterogeneous nature. Indeed, systematic differences can occur due to site-specific conditions or due to bias in population characteristics. Moreover, some tasks are difficult to generalize from one dataset to another, since not all datasets are consistently labeled, or are labeled at all. As such, Kushibar et al. propose a transductive transfer learning approach for domain adaptation to reduce the domain-shift effect in human brain MRI segmentation. The transductive property means that there are two disjoint source and target domains, where label annotations are only available in the source domain, but examples from the target domain are also present. The proposed network is jointly optimized by integrating both source and target images into the transductive training process, minimizing the domain-shift effect with a histogram loss at feature level. The method shows performance improvements up to 10% in terms of Dice similarity coefficient for the segmentation of subcortical brain structures and white matter hyperintensities, compared to a model pre-trained in the source domain only.

Meyer et al. propose to increase the generalization capability of state-of-the-art convolutional neural network (CNN) models trained on homogeneous datasets by applying an intensity-based data augmentation approach based on Gaussian mixture modeling. This approach is shown to significantly impact the generalization performance of brain structures segmentation when the training set is very homogeneous, but also when it consists of heterogeneous multi-scanner brain images.

Valverde et al. employ CNNs for the task of lesion segmentation in rodent brains. They suggest that an architecture resembling an autoencoder has better performance compared with three other convolutional neural networks specifically designed for medical image segmentation. Moreover, when comparing versions trained on homogeneous and heterogeneous training datasets, it is clear that increasing training data diversity improves performance, as measured by the capability to extrapolate to different-looking ischemic brain lesions.

Not only combining datasets, but also combining existing modeling methods can be beneficial for obtaining more robust biomarkers. This is illustrated by Lu et al., who focus on quantitative diffusion measures for classifying multiple sclerosis lesions. They use eight open-source biophysical models of multishell diffusion data to reconstruct the isotropic and intra-axonal compartments, and identify the microstructural diffusion measures that are most discriminative for focal pathology. Further, they show that some of the combinations of the selected normalized diffusion measures better correlate with patients' disability and neuroaxonal damage than individual measures.

While deep learning techniques play an important role in current neuroimaging research due to their ability to increase reliability of computed biomarkers, there are still essential gains to be made by improving MRI acquisitions and parameter map reconstructions. For instance, Emmenegger et al. assess the effect of radio-frequency transmit (B1+) field inhomogeneities correction on the accuracy of MR G-ratio weighted imaging, which is an aggregated measure of relative myelination of axons across the entire brain white matter. B1+ correction *via* a measured B1+ field map is the method of choice to reduce bias and test-retest error. However, if the B1+ field map cannot be acquired, a data-driven B1+ correction approach is also proposed, and shown to reduce the error and bias by a factor of three.

Metin and Gökçay highlight the value of using directional information from diffusion tensor imaging of the brain for group statistics, rather than scalar metrics that consider only the magnitude of the diffusion. A typical scalar metric used in group studies is the Fractional Anisotropy map. Directional statistical analysis is particularly important along the white matter tracts, especially when the tract length increases.

Barakovic et al. propose a more robust estimation of the axon diameter index of pathways by jointly estimating the microstructure properties of the tissue and the macroscopic organization of the white matter connectivity. The method overcomes limitations of previous voxel-wise approaches, which neglect the fact that axons are continuous three-dimensional structures that are not limited to the extent of each voxel. By computing the axon diameter index in bundles of streamlines, where each streamline represents a group of axons that share a similar trajectory, the method is able to estimate an average diameter for the represented group of axons. As such, they show that the fiber bundle composition agrees with histology and known anatomy.

The value of brain imaging biomarkers is crucial since early stages of development. Thus, imaging the neonatal brain with the aim of establishing patterns of (normal or abnormal) brain development is also an important and very active topic of research. It is now possible to acquire high resolution (isotropic 0.4 mm) images in a short time (6 min), due to novel super-resolution reconstruction of three short duration scans with variable directions of slice selection (Sui et al.).

Uus et al. highlight the importance of employing a suitable atlas of normal neonatal brain development. They build a single multi-channel spatio-temporal atlas based on multiple metrics extracted from both diffusion and structural MRI, and then compare, in this novel atlas space, two groups of neonates: born at term and preterm. Significant effects linked to prematurity are shown to be present in multiple brain regions, including the transient fetal compartments, indicating that white matter maturation is altered by preterm birth.

Grigorescu et al. predict tissue segmentation maps of neonates on T2-weighted magnetic resonance imaging data. Similarly to Kushibar et al., they employ domain adaptation techniques for the challenging task of brain segmentation in a preterm-born neonatal population, where the training set consists of an annotated dataset acquired from term-born neonates with a different scanner and acquisition protocol. Importantly, adding the domain adaptation to the model did not degrade performance in the source domain. Moreover, in line with Meyer et al., the authors show the importance of adding data augmentation during training.

Finally, neuroimaging methods should not only act as tools for new scientific discoveries, but should also provide practical solutions for different neurological conditions in clinical practice. As such, La Rocca et al. develop a novel approach based on structural MRI to analyse interactions between brain components. Based on these novel features, they train a classifier able to predict with an accuracy of 70% whether subjects who suffered a mild traumatic brain injury will have at least one seizure in the future. Princich et al. revisit known MRI volumetry biomarkers that can assist in the diagnosis of temporal lobe epilepsy. Despite observing differences between different MRI segmentation software in terms of hippocampal volumetry (here, FreeSurfer and volBrain are compared), the study strongly reinforces the value of hippocampal asymmetry in differentiating healthy controls and epilepsy patients with hippocampal sclerosis. Bai et al. focus on childhood obstructive sleep apnea, a sleep-related breathing disorder that can have an important negative impact on neurological development. Functional MRI reveals altered spontaneous brain activity, with dysfunctions occurring in the default mode network, the frontal lobe, and the lingual gyrus.

In conclusion, this Research Topic clearly illustrates that the field of computational neuroimaging is active and fascinating, with a wide range of novel methodologies aiming at reliability and generalizability through domain adaptation, data augmentation, super-resolution, and quality control. Important applications presented here aim at understanding brain development, connectivity and microstructure, as well as brain diseases such as multiple sclerosis, epilepsy, post-traumatic brain injury, and sleep dysfunction.

## Author Contributions

DMS wrote the draft of the manuscript. MBC, TBD, and KVL contributed to manuscript revision, read, and approved the submitted version. All authors contributed to the article and approved the submitted version.

## Conflict of Interest

DMS was employed by icometrix. The remaining authors declare that the research was conducted in the absence of any commercial or financial relationships that could be construed as a potential conflict of interest.

## Publisher's Note

All claims expressed in this article are solely those of the authors and do not necessarily represent those of their affiliated organizations, or those of the publisher, the editors and the reviewers. Any product that may be evaluated in this article, or claim that may be made by its manufacturer, is not guaranteed or endorsed by the publisher.

